# HIV Risk Behaviors among African American Women with at-Risk Male Partners

**DOI:** 10.4172/2155-6113.1000221

**Published:** 2013-07-25

**Authors:** Keisha C. Paxton, John K. Williams, Sherica Bolden, Yesenia Guzman, Nina T. Harawa

**Affiliations:** 1 Department of Psychology, California State University, Dominguez Hills, USA; 2 Department of Psychiatry & Biobehavioral Sciences, Semel Institute for Neuroscience & Human Behavior, University of California, Los Angeles (UCLA), USA; 3 JWCH Institute, USA; 4 Department of Research/Medical Sciences Institute, College of Medicine, Charles R. Drew University of Medicine and Science, USA

**Keywords:** African American women, Sexual behavior, HIV/AIDS, Prevention

## Abstract

**Background:**

HIV continues to impact African American women at alarming rates. Yet, few researchers have examined the relationship factors promoting unprotected sex within African American communities, especially instances in which women are aware that their male partners are engaging in high risk behaviors. This qualitative study explored the sexual behaviors, relationship characteristics, and HIV prevention strategies utilized by African American women in relationships with African American men at-risk for HIV.

**Method:**

To understand the issues that should be addressed in a sexual risk-reduction intervention, data were collected from three, two-hour focus group discussions (n=24) comprised primarily of low-income African American women with histories of at-risk male sex partners. At-risk partners included specifically men who had sex with other men or with transgender individuals, used crack cocaine or injection drugs, had lengthy incarceration periods, or an unknown sexual history. Discussion questions examined external factors affecting sexual risk behaviors such as societal pressures, peer norms, and financial vulnerability. Discussions were audiotaped, transcribed, and analyzed using a consensual qualitative research approach.

**Results:**

Five themes, including self-esteem, social influences on behavior, relationship fidelity, sexual risk behavior, and partners' sexual behaviors, were identified as placing women at increased risk for HIV. Reasons for inconsistent condom use included concern for maintaining the relationship and substance use before and during sex. African American women also believed that men who have sex with men and women (MSMW) were dishonest about their sexuality due to stigma towards homosexuality/bisexuality. Despite these challenges, participants indicated that African American women have a strong sense of pride that can positively impact behaviors in relationships.

**Conclusion:**

The findings of this study support that social and contextual factor such as emotional and financial issues, culture, history, and relationship dynamics need to be considered when developing tailored sexual health interventions for this population.

## Introduction

HIV/AIDS continues to be a health crisis for African Americans, with women being significantly impacted. Heterosexual contact is the primary mode of transmission for African American women [1], thus there is a need to examine the emotional, historical, and sociocultural factors promoting unprotected sex among this population [2]. Both media and public health attention to this problem has increased in recent years. Nevertheless, many heterosexual African American women are not concerned about contracting HIV, nor do they perceive it to be a major health risk directly affecting them [3,4]. One prominent risk for African American heterosexual women is the risk behaviors of their male partners. It is known that African American heterosexual women prefer African American male partners [5]; rates of HIV are high among African American men [1], as well. Risk behaviors, such as concurrent sexual relationships and sex while under the influence of drugs and/or alcohol, in male partners who have sex with both men and women place African American women at risk for HIV/AIDS [6].

Despite knowledge of their partners’ concurrent relationships, some African American women continue to engage in unprotected sexual intercourse [2,7]. Some report difficulty asserting condom use [8]; while others prefer not to use condoms within ongoing relationships [2,7]. Many women also reported the use of drugs and alcohol during sex even though they were aware that this heightened their risk for HIV infection [9,10]. It is important to address the power dynamics that impact condom use.

Issues related to power and gender inherently impact condom usage in male-female sexual encounters. Men primarily have control over condom use in heterosexual relationships [11], and women, experiencing a lack of power, may have difficulty negotiating condom use [12]. Furthermore, it may be especially important for African American women to allow their male counterparts to have control over condom use in order to maintain gender roles, their own sense of femininity, and intimate relationships [11]. For example, African American women may allow their male partners greater control in relationships in order to highlight their masculinity and counter the lack of control that their partners may experience in society as African American men [11]. Additionally, sexual intercourse sans condoms may imply a greater form of intimacy, trust, and/or validation of the relationship status [13]. Relationships that are perceived to be monogamous create a sense of security for women that may actually make them more susceptible to HIV and other sexually transmitted infections (STIs) by leading to less consistent condom use [9,10]. Pressure to engage in sex without using a condom may also be exacerbated by the women’s belief that they must compete with one another in order to *keep a man* [2].

There are many sociocultural and contextual factors that can influence sexual behavior [3]. Racism, socioeconomic status, and interpersonal power influence susceptibility to engaging in risky behavior [14]. Research about condom use among African Americans has shown mixed results. Whereas some studies suggest low levels of condom use and negative attitudes regarding condoms among African Americans [3,7], other studies suggest that African Americans use condoms as much as or more than their counterparts from other racial/ethnic groups [15,16]. Furthermore, there also exists an imbalance in the male-to-female sex ratio among African Americans [17,18] resulting in a shortage of single, heterosexual African American men and the aforementioned pressure that results from competition among women.

Though individuals are more likely to report using condoms with a secondary partner than with a primary partner, many still report not using condoms at all [19,20]. Such overlapping sexual partnerships have potential health consequences such as more rapid spread of HIV and other STIs. HIV prevalence is elevated among individuals reporting sex with a secondary partner [21] or a partner who had secondary partners [22]. African American men are much more likely to engage in extramarital relationships than African American women [21,23,24], which supports the likelihood that women’s greater vulnerability to HIV infection is attributed more to their partners’ higher risk behavior than their own [25].

In order to better understand the HIV risk factors of African American women, it is critical to address the HIV risks behaviors of the African American male-female dyad. African American men who have sex with men (MSM) or with both men and women (MSMW), have the highest rates of HIV/AIDS in any racial/behavioral risk group in the U.S. [1,26]. Also, African American MSM are more likely to report bisexual behavior than any other group of MSM [27–29] and the least likely to discuss their bisexual behavior with others [30]. MSM who self-identify as “on the down low” are less likely to obtain HIV testing than are those MSM who do not [31].

The “down low or DL” term that was previously used to describe behaviors and activities that should be kept secret [32–34], has been adapted and perpetuated by the lay community and media to describe African American men who identify as heterosexual and who do not disclose their same sex behaviors with female partners [35]. Understanding the meaning of identifying with the down low or DL term may be important for assessing sexual risks. While the lay media has suggested that men on the DL may be vectors of HIV to male and female partners, some research suggests otherwise. In a study conducted by Bond et al., down low identity was not associated with greater sexual risk behaviors with male or female partners when comparing behaviors of DL identified and non-DL identified MSM [32]. Importantly, identifying as DL did not necessarily mean having female partners, as half of the DL-identified participants did not report recent female partners. This underscores the importance of behavior rather than identity labels in understanding behavior dynamics and risks in populations. It is also worth noting that social factors such as financial vulnerability and gender role perceptions have been found to influence whether women remain in sexual partnerships with men that they know to be at risk for HIV [35].

Given that sexual decision-making is influenced by emotional, sociocultural and/or historical factors and executed at the individual (male or female) or dyadic levels, understanding what contributes to sexual risk taking for African American women with African American male partners is essential to addressing the HIV epidemic among this population. The current study will inform HIV/STI prevention and testing programs that specifically address issues pertaining to African American women whose sexual behaviors and partnerships may put them at risk for HIV/AIDS and STIs. We collected qualitative data from three focus groups of African American women in Los Angeles. We analyzed the transcripts of these discussions in order to better understand the knowledge and attitudes regarding HIV/STI risk behaviors and partner risk factors among African American women.

## Method

### Participants

Between January and May 2008, 24. African American women living in Los Angeles, California were recruited to participate in one of three focus groups. These focus groups were approved by the Institutional Review Boards at both UCLA and Charles R. Drew University of Medicine and Science as part of the formative research for the development of a sexual health intervention for African American women with *at-risk* male partners. To be eligible for participation, women had to self-identify as African American, be 18 years of age or older, speak English, and report a history of sexual activity with an at-risk African American male partner. *At-risk* was defined as a man who had at least one of the following known or suspected behaviors/ experiences: 1) sex with men or with male-to-female transgenders; 2) injection drug use (IDU) or crack cocaine use; 3) incarceration greater than 6 months; or 4) unknown sexual history (“a male partner whose sexual history you did not know”).

Participants were recruited via street outreach and through referrals from local health service organizations. The study received 46 calls with 42 of the women being eligible for the study. Among the eligible women, 24 (57%) participated in one of the three focus group discussions, each of which lasted between 100 and 124 minutes. Eligible participants were between 26 and 54 years of age with a mean age of 42 years. Seventy-one percent had a monthly individual income of less than $1000. The majority of the participants had a high school education or less (93%), were single (82%) and lived alone (76%). Seventy-one percent of the participants reported having a regular male partner; 36% reported not knowing their partner’s sexual history; 10% reported knowledge of their partners having sex with men, and 29% and 28% *suspected* their partners of sex with men or male-to-female transgenders, respectively. Thirty-eight percent knew and another 10% suspected their partners of having an incarceration history of greater than six months. Thirty-six percent of participants reported crack cocaine use and 23% suspected crack cocaine use by partners; 14% suspected partners of injection drug use. Sixty-eight percent reported no condom use in the prior three months.

### Measures

A semi-structured interview guide was developed for the focus groups by the study’s principal investigator and co-investigators, with input from a community advisory board comprised of members of the target population and service providers to this group. Questions were also developed and selected based on a review of the literature on African American female sexuality, HIV risk behavior, and male-female sexual relationships. Focus group questions were primarily open-ended. Questions explored experiences of being an African American woman, the influence of Black culture on sexual decision making, the use of condoms, the implications of having both primary and secondary sexual relationships, and perceptions of African American male sexual behaviors.

### Procedures

Individuals interested in participating in the study contacted study personnel and were screened for eligibility via telephone. If they met the inclusion criteria, the study was explained to them and they were invited to participate in a focus group. Three separate focus groups were conducted. Two groups were composed of women who had not participated in our community partner’s existing HIV-prevention program, entitled *Healthy Alternatives to Reduce the Risk of HIV Program* (HARRP; n=6 and n=9), while one group was composed of women who had participated in this program (n=9). HARRP participants had only to report sex with an at-risk male in their lifetime, since they would potentially have decreased their HIV risk because of their participation in HARRP. All other participants had to report sex with an at-risk partner in the prior three months. The reason for focusing on these groups was to include the opinions of women with little sexual risk-reduction information, as well as those who had received in-depth information through a community program. Focus groups were conducted at community agencies in the Los Angeles area. Participants were not required to be affiliated with the agencies. Upon arrival to the focus group location, informed consent was obtained from each individual. Focus groups were facilitated by two African American study co-investigators/authors, a male (psychiatrist) and female (psychologist) dyad. Upon completion of the group discussions, participants were asked to complete a brief anonymous demographic questionnaire. Focus groups were audiotaped and transcribed verbatim by a professional transcriptionist. Participants were compensated $25 for their participation.

A grounded theory approach was used to guide data analysis [36]. The four-member team analyzing the data consisted of one of the co-investigators who had co-facilitated the groups, a graduate student, and two undergraduate students. Each member of the team initially read each transcript, then engaged in a second read to analyze for themes. Two of the members of the team coded themes separately, then compared themes and produced one set of themes via a consensual qualitative research approach [37]. A third team member separately produced themes and then compared those to the initial set.

## Results

In general, there were no significant differences discerned between the HAARP and NonHARRP focus group discussions of their sexual experiences and opinions of African American women’s behaviors. On a few occasions, members of the HAARP focus group referred to having knowledge of STIs and/or HIV transmission from their participation in the HAARP intervention. Common themes across these two sets were selected as the final group of themes. Themes were allocated into the following five categories: (1) African American women’s self-esteem, (2) social influences on behavior, (3) sexual risk behavior, (4) relationship fidelity, and (5) male partners’ sexual behaviors.

### African American women’s self esteem and influences on relationships and behaviors

The focus group participants indicated that African American women’s views about themselves had the potential to both positively and negatively impact their behaviors, particularly within relationships. For example, they expressed genuine pride in being African American women. “God’s been there with me and for a Black woman; I understand what my ancestors, what all they went through and all that. You know what I’m saying? And I’m very blessed and proud to be Black” [NonHARRP, 47 years old, not married, regular/main partner; partner’s risk category: unknown sexual history]. This pride, however, comes at a price. Participants reported that there were higher expectations for African American women in that they were expected to be “superwomen,” flawlessly taking care of house and home. The participants also expressed the feeling that African American women must demand respect in order to receive it, unlike their white counterparts.

*For me, as a Black woman, I have to have some respect for myself. Okay? …Being a Black woman, I have to have some respect for myself because for me, I attract who I am and if I act like a slut, I’m gonna be treated like a slut* [NonHARRP, 39 years old, married and living with partner; partner’s risk categories: unknown sexual history and crack cocaine use].

With the burden of demanding respect also comes the need to respect oneself. It appears that self-respect is sometimes a challenging battle.

*…I’m just at a point now because I had ten years clean and sober at one time, and I had to get to a point where I didn’t want to be promiscuous and stuff like that. I just started respecting myself because I know—and it’s the truth, because I didn’t need this. I couldn’t even see that the way I treat myself is how a man would treat me* [NonHARRP, 39 years old, married and living with partner; partner’s risk categories: unknown sexual history and crack cocaine use].

### Social influences on behavior

Participants reported conflicting feelings about communal influences on their relationships and sexual behaviors. On one hand, they reported that their families, church, and friends could impact their behaviors. The following illustrates the influence of the church and friends on one participant who had been involved in sex work.

*But I want to stress on the community and the church thing… I used to work on the Boulevard. Church that I had been in for almost four and a half years now, they saw me out there. They saw me doing my thing. And I went to this church and I met a few people with the trust issue… But I was drawn to these people. And so I would still go out there and do my thing, but it’s hard to do your thing when you’ve got to be careful which street you’re on because so-and-so lives on this street* [HARRP].

On the other hand, participants also stated that social forces had no impact on their behaviors and they would make their own sexual decisions despite what family and friends felt. This was illustrated by the following participant’s response when asked about how family or social structures (e.g., church) impacted her behavior: “What my family thinks really doesn’t matter at all because I have pretty much learned how to pull myself up from my own boot straps” [NonHARRP, 26 years old, not married, no regular partner; partner’s risk categories: sex with men, sex with male-to-female transgendered individuals and crack cocaine use].

### Sexual risk behavior

The theme of sexual risk behavior was described as involving three different but important issues: condom use, sexually transmitted infections, and substance abuse. With respect to condom use, participants reported that African Americans use condoms inconsistently and that risk status of self or partner does not necessarily impact whether or not they use condoms in relationships.

*I was real young and stupid…I accepted so much B.S. that I even seen a girl comes over there. He had both of us there at the same time… And that didn’t make me go use no condom* [NonHARRP, 39 years old, married and living with partner; partner’s risk categories: unknown sexual history and crack cocaine use].

*My relationship with my friend has been going on, like, three or four months and we haven’t used a condom yet* [NonHARRP, 47 years old, not currently married, no regular/main partner; partner’s risk categories: sex with men, sex with transgendered individuals, incarcerated for more than six months, and injection drug and crack cocaine use].

*My second husband was bisexual, I guess because I didn’t see him outside of jail [with men], but I know that inside of jail—not only that but when he was in his addiction, I know he was out there selling himself or had encounters with men that would pay for it. But I didn’t use condoms when [I was] up with him* [HARRP, 54 years old, not currently married, no regular/main partner; partner’s risk categories: sex with men, incarcerated for more than six months and crack cocaine use].

A related issue was the expectation that their male partners should use condoms with their secondary partners so as not to bring home any diseases; however, not using condoms was seen as a “privilege” bestowed upon the primary female partner. One NonHARRP participant stated, “He can use a condom with *her*.” Participants seemed somewhat split on the topic of condom use, with some believing that most African Americans use condoms and other believing they do not. Age appeared to be the factor contributing to the difference in opinions, such that participants believed younger African Americans (i.e., adolescents and young adults) tended to use condoms more than older African Americans did. These participants based their opinions of condom use among younger African Americans through their experiences with their own children.

Sexual risk behavior, as it concerns STIs, emerged as an interesting area with conflicting perspectives. Participants reported that either they or their partners would engage in unprotected sexual behavior while knowingly having an STI. They reported that this type of behavior would most likely occur if one were under the influence of drugs or exchanging sex for drugs or money. Respondents also stated that African American women were more likely than their male counterparts to reveal their HIV serostatus. Additionally, participants also reported that for some African American women, both abstinence and condom use (including the female condom) were good options for protection from pregnancy, HIV and other STIs. One HARRP participant expressed, “I suggest if you don’t want kids and the man don’t want to use a condom, stay celibate. Stay to yourself. Don’t do anything. Just stay abstinent.”

A small subset of participants reported engaging in group sexual activities (i.e., “orgies”). One participant admitted to having sex in the same room as another couple and then later all four individuals engaged in sexual activities together. Another participant suspected her son of having group sex sessions at her home. Although participation in group sex did not appear to be a common activity among this sample, it is important to acknowledge this phenomenon as it is a high risk behavior.

Substance use appeared to have a significant impact on condom use and health protective behaviors. Participants reported that substance use was not uncommon in the African American community. In addition to alcohol and marijuana use, participants reported that it was not unusual for African American women to use crack, ecstasy, and methamphetamines. One HARRP participant stated, “I did more promiscuous stuff and dangerous stuff more with meth than with any other drug.” Additionally, participants reported inconsistent condom use as being particularly problematic while intoxicated.

*You know, some women when they’re in their addiction, they get very promiscuous and they do what they got to do. And they don’t use condoms, you know, they either don’t have one, they’re not thinking about one, they don’t care about one, you know, and so it’s mainly women in their addiction and I know when I was in mine, I did little things like that*. [NonHARRP, 47 years old, not currently married, has regular/main partner; partner’s risk categories: unknown sexual history].

Participants also stated that it was fairly common for African Americans to use substances prior to and/or during sexual activity and that it enhanced their desire for and enjoyment of sex.

*…But as soon as I got that crack, I was enjoying him, and when the crack was gone, ‘Get up off me.’…It’s the crack or whatever the drug is at the time* [HAARP].

In other instances, participants discussed themselves and other African American women trading sexual favors for drugs or money to purchase drugs, even if the sex was not part of what they desired.

*So I put myself at risk [to get drugs]. Right now I’m hurting. I want these drugs. What you gonna give me? Let’s do this and get this over with. And I pray to God, ‘Please don’t let me have nothing’* [HAARP].

### Relationship fidelity

Fidelity was a category that included conflicting themes. Participants reported that infidelity might be acceptable under particular circumstances, such as their partners using condoms or their partners being discreet about their behaviors with secondary partners. One participant stated, “I would prefer him to come home and say, ‘Let’s use some condoms,’ than to bring something home” [NonHARRP, 50 years old, married and living with partner; partner’s risk categories: sex with men and crack cocaine use]. For some, the realization that a partner might not be faithful and also not use condoms with their other partners was more difficult to deny:

*But I allowed [him] to have sex with the guy that I originally was starting to date just to see him get his pleasure that I wasn’t able to do. And I thought he would put on a condom, but he didn’t*. [HARRP, 41 years old, not married, has a regular/main partner; partner’s risk categories: sex with men, sex with transgendered individuals, incarcerated for more than six months, injection drug use and crack cocaine use].

The challenge of introducing condoms, as well as reintroducing condoms, came up as a theme. It was thought that introducing condoms into an established relationship might cause suspicion on the part of the male or female partner. Nevertheless, there was still a potential relationship risk involved in trying to protect one’s primary partner through condom use with a secondary partner. For example, one NonHARRP participant stated, “If I find a condom in my old man’s pockets or in my house somewhere, we going to tear the house up.” Other women’s quotes also illustrated concerns about partner fidelity, fear of losing their partners and their relationships, and a willingness to engage in behaviors that they did not enjoy in order please their partners:

*…I feel if he had to go outside our relationship, I’m not doing something right* [NonHARRP].

*We could all say it here that we use condoms, that we’re protected and stuff like that, but a lot of times we wasn’t. Well, I’m gonna speak for me. You feel me?…And like, even they say even when you – I mean, I’ve been married for, like, almost 11 years, and some people say that we should still use condoms…No, we don’t*. [NonHARRP, 39 years old, married and living with partner, has a regular/main partner; partner’s risk categories: unknown sexual history and crack cocaine use].

*…For your satisfaction, whether I want to participate in the act or not. But to please that person, I participate anyway…*[HARRP].

Finally, whether or not a woman has a child with her partner might lead to enduring an unhealthy relationship with him. The following comments illustrate how such choices were influenced both by familial norms about the expectations of women as mothers and by their own gender-based experiences as children:

*Five kids and five daddies. One daddy I stayed with because, even though he was beating the shit out of me, my grandmother said, ‘There’s just one thing about it baby. That’s your baby’s daddy. You’ve got to make it work for that baby. …That’s the child’s father and you’re supposed to be with him’* [HARRP, 43 years old, not married, has a regular/main partner; partner’s risk categories: sex with men, sex with transgendered individuals, incarcerated for more than six months, and crack cocaine use].

*For me, it was more like a world without my father, not knowing him. So when I had my two sons, to me it was important that they needed their father. And I hung around until he passed away so they would at least know their father, at least know the other side of them, his family* [HARRP].

### Male partners’ sexual behaviors

The issue of sexual orientation and behaviors of male partners emerged as a theme and highlighted the lack of acceptance of gay and bisexual men by the African American community. Although many participants reported having gay men as friends, they indicated that some African American men who engage in same-sex sexual behaviors feel ostracized by the African American community. Participants explained that many African American men may not disclose their sexuality and engage in covert same-sex relationships while maintaining relationships with African American women. This then provides a “cover” to give the appearance of heterosexual behavior. Another issue was greater acceptance of infidelity if a partner cheated with a woman rather than with another man. One participant stated, “It can make it worse if it was a man. If it’s a woman, I can get over it” [NonHARRP, 50 years old, married and living with partner; partner’s risk categories: sex with men and crack cocaine use]. Another participant expressed the emotional impact of learning that her partner engaged in sex with men as follows:

*Then when he finally broke down and told me the truth, I said, ‘It ain’t gonna make me love you any less.’ And it just happened the guy smoking was gay. But I allowed [him] to have sex with the guy…And he enjoyed it so much that I sat over there in the midst of hitting this pipe crying because I felt left out and cheated. You know what I’m saying? But it took that for me to see that men don’t always come and tell you what you think you should know*. [HARRP, 41 years old, not married, has a regular/main partner; partner’s risk categories: sex with men, sex with transgendered individuals, incarcerated for more than six months, injection drug use and crack cocaine use].

Participants also reported that they believe that women will know if their partners are engaging in sex with other men -- “Me, myself, being a Black woman, I would know” [NonHARRP]. Participants implicated incarceration as a major contributor to same-sex sexual behavior among African American men, and that this is what caused them to become feminine, to desire sex with men, or to prefer anal sex post release. One woman expressed, “…I had a boyfriend and he went to jail. When he came home, something just told me that he just seemed like he was more woman than me” [NonHARRP, 50 years old, married and living with partner; partner’s risk categories: sex with men and crack cocaine use]. Another participant stated, “When they go to jail or go to prison, the first thing they want to do—‘Can you turn over and I get you from behind?’ and I’m like, ‘You’ve been to jail, huh?’” [NonHARRP, 52 years old, not married, has a regular/main partner; partner’s risk categories: unknown sexual history]. Regardless of his incarceration history, other participants also echoed the sentiment that requests for anal sex were clues that their partners might be “gay”.

*I had a situation that I was with a man, and he kept on asking me for anus sex. And I was like, ‘No, I don’t do that.’ And every time we have sex, that conversation came up. And something just in the back of my head, like you, said, “Is this man gay?” And I asked him. And he told me, ‘Yes, I’ve been with a couple of men.’ And I was like, ‘Why didn’t you tell me this?’ So I started using rubbers with him because I was scared. Then I went to go get my test done, and then I left him* [HARRP, 44 years old, married and living with partner; partner’s risk categories: unknown sexual history, sex with men, and incarcerated for more than six months].

## Discussion

This study sought to gather current data from African American women with histories of sexual relationships with at-risk African American male partners and to compare the findings to previous researchers who studied African American women’s risk behaviors, attitudes and perceptions at an earlier point in the HIV epidemic [7,8,14]. Despite the heightened attention that has been brought to the burden of HIV in African American communities and to African American men who have sex with both men and women, many African American women continue to engage in unprotected sex with potentially risky men for reasons similar to those described in earlier studies [7,8,14]. Our findings indicate that targeting motives and creating incentives for African American women to consistently engage in positive sexual health behaviors would require engaging a complex array of both social and contextual factors, including emotional issues, culture, history, and relationship dynamics.

### The importance of relationships

A common theme in this study, as well as in the literature [38] on African American women, was the pride that many African American women wear as both a “crown and a crutch.” The stereotype of the “strong, African American woman” can contribute to a superwoman complex, perhaps making them more vulnerable to unhealthy behaviors in sexual relationships. Participants reported a willingness to accept unfaithful partners due to loneliness and the desire for a relationship. This may be related to the statistics that appear to put African American women at a disadvantage in relationships. African American women outnumber African American men 10 to nine [39], and 65% of African American single mothers report having never married [40]. In light of these statistics, it is necessary to acknowledge the importance and challenge of being in a relationship with a man to many African American women, while helping them to take on protective health behaviors. Additionally, the meaning of a relationship needs to be explored within the sociocultural context of African American women. In our sample, relationships for many women meant security, a source for intimacy, and a source of resources. For example, being with a male partner who was unfaithful was sometimes overlooked when they provided other benefits such as being a father to their children or contributing income to the household [35]. Wyatt [41] suggests her Sexual Health Model (SHM) as a culturally appropriate theoretical perspective from which to understand and address sexual risk behaviors among African American women. She states that many African American women may operate in a *survival mode* in which coping and addressing one’s needs for survival may take priority over addressing sexual health behaviors.

### Challenges with condoms

A challenge for interventionists is teaching African American women how to introduce condoms into a relationship. Understanding the socio-cultural context of the woman’s life and relationship is necessary [42]. The principle of least interest [43] suggests that African American men have ample opportunities for intimate relationships and as such, may be less interested in any one particular relationship, thus giving them greater power in heterosexual relationships. This power imbalance can contribute to African American women’s decision to not assert condom use. Oddly enough, women may not be aware of the power they possess. African American men may place the decision to use condoms in the hands of their female partners and follow the woman’s lead, only using condoms when she brings it up [44]. However, being in a relationship may be perceived as more important and/or necessary than using condoms to protect one’s health. For example, the economic advantages of being in a relationship may also outweigh the health protective benefits that condoms bring [45].

Participants in the current study reported that if they or their partners introduced condoms into an established relationship, it would breed suspicion of infidelity. A woman who is concerned about her partner’s fidelity may fear losing her partner due to acting on her own concerns for protecting herself from an STI. Furthermore, there was often an unstated agreement that male partners would use condoms with secondary partners and save skin-to-skin contact, which was perceived as a more intimate activity, for the primary partners. Additionally, some women feared that condom use with a primary partner would subversively imply permission to engage in other sexual relationships, as suggested by Nunn and colleagues [45] in their qualitative study of African American women. It is critical to balance and address priorities among these potentially competing issues in a culturally sensitive manner.

Consistent with other research [24], our study suggests that younger African American women may be more likely than other African American women to use condoms. This may be explained by a cohort effect reflecting the impact of growing up in the age of HIV/AIDS; the younger generation of African American women may feel a greater sense of empowerment with respect to condoms and sexual health than their older counterparts. Alternately, they may be less likely than older women to be engaged in serious partnerships wherein condoms are used less often because they present many of the challenges described above.

### Community norms and influences

Respondents in this study discussed same-sex sexual behavior among male partners as an issue faced by some African American women, placing them at increased risk for HIV. Some participants blamed the African American community for African American men engaging in secret sexual liaisons with men, as gay men have historically been ostracized by such institutions as the African American church [46]. Participants felt that the fear of being ostracized contributed to the so-called “down low” phenomenon of African American men engaging in relationships with women openly and men secretly. Research has shown conflicting findings in regard to DL-identified and/or MSMW engaging in greater sexual risk behaviors than heterosexual or non-DL identified MSM [32,47]. More intervention and research focusing on helping these men minimize the risk of HIV and STIs to themselves and their partners is still needed.

Findings from this study suggest that social influences such as the church, family, and friends are potential intervention points for protective sexual health behaviors but must be considered carefully. Participants reported that these social influences may impact their behaviors; however, for some, they also stated that they make their decisions independently of what others think. Herein may enter the impact of being a proud African American woman. The stereotype of African American women as superwomen could lead to perceptions of having to do it all and to doing it all alone. Thus, African American women may not want to feel “helped” by community members, as this may be interpreted as a weakness. To aid in health interventions, a useful approach may be to underscore to African American women the benefits of protective health behaviors that allow them to remain healthy and thereby, strong. In doing so, they would then be able to assist and support their communities (i.e., children, partners, families), which would be congruent with the strong, independent image of African American women. Furthermore, women would not be threatened by family and friends offering them assistance or advice or potentially perceiving them as being weak.

### Limitations

As with all research, social desirability may pose a limitation. This is particularly true with qualitative research on a topic as sensitive as sexual behavior. It may be that some participants withheld pertinent information due to embarrassment or to save face. Nevertheless, participants in these focus groups appeared to be very forthcoming about their sexual histories and opinions of sexual behaviors among African American women. The results of this study should be understood in the context of the study sample. The majority of participants reported a history of homelessness and drug use and some also reported experiences with incarceration. This sample represented an at-risk group of African American women whose experiences may reflect those most susceptible to maladies related to sex with at-risk male partners.

Importantly, while this study focused on the risk behaviors of the male partners, it is critical to realize that the women also had their own risk behaviors. As evident from the quotations, many engaged in multiple and concurrent partnerships, exchange sex, and drug use. Also, having modest assets in the form of education, housing and income may have added to the vulnerability and potential risks that they were willing to endure. Again, these findings highlight the need to examine risk behaviors at the individual and couple level and within the context of the women’s lives.

## Conclusions

African American women are a complex heterogeneous group with ongoing, elevated risk for HIV and sexually transmitted infections despite increased attention to the HIV epidemic in African American communities. Tailored interventions addressing emotional, sociocultural, and historical issues are imperative to support the sexual health of this population. This study provided important information from at-risk African American women that elucidates factors influencing sexual behavior. Although this information cannot be generalized to address the needs of all African American women, it provides useful information for the development of culturally tailored sexual health interventions for this population.
